# Effects of Individual and School-Level Characteristics on a Child’s Gross Motor Coordination Development

**DOI:** 10.3390/ijerph120808883

**Published:** 2015-07-30

**Authors:** Raquel Chaves, Adam Baxter-Jones, Thayse Gomes, Michele Souza, Sara Pereira, José Maia

**Affiliations:** 1 Academic Department of Physical Education, Federal University of Technology, Paraná (UTFPR), Av. Sete de Setembro, 3165, 80230-901-Curitiba/PR, Brazil; 2 CAPES Foundation, Ministry of Education of Brazil, SBN Quadra2, Bloco L, Lote 06, 70040020, Brasília, Brazil; E-Mail: mcsouza85@hotmail.com; 3 College of Kinesiology, University of Saskatchewan, 87 Campus Drive, Saskatoon, SK S7N 5B2, Canada; E-Mail: baxter.jones@usask.ca; 4 CIFI^2^D (Centro de Investigação, Formação, Inovação e Intervenção em Desporto), Kinanthropometry Lab, Faculty of Sport, University of Porto, Rua Dr. Plácido Costa, 91, 4250-Porto, Portugal; E-Mails: thayse_natacha@hotmail.com (T.G.); sara.s.p@hotmail.com (S.P.); jmaia@fade.up.pt (J.M.)

**Keywords:** motor coordination, hierarchical linear modeling, schoolchildren

## Abstract

The aim of this study was to identify child and school-level characteristics that explained inter-individual differences in gross motor coordination (GMC). Participants (n = 390), recruited from 18 Portuguese primary schools, were aged 6 to 10 years of age. Birth weight, body fat (BF), physical activity (PA), physical fitness (PF) and GMC were assessed. School size, setting, infrastructure and physical education classes were considered as school context markers. A multilevel modeling approach was used to identify hierarchical effects (child and school levels). It was found that children-level variables (sex, PF, and BF) significantly explained 63% of the 90% variance fraction at the individual level; boys outperformed girls (*p* < 0.05), individuals with higher BF were less coordinated (*p* < 0.05), and those with higher PF were more coordinated (*p* < 0.05). School-variables (e.g. school size and playing surface) explained 84% of the 10% variation fraction. These findings confirm the roles of sex, PFS and BF. Interestingly they also suggest that the school environment plays a minor but significant role in GMC development. However, it is important to stress that the school context and conditions can also play an important role in a child’s motor development, providing adequate and enriching motor opportunities.

## 1. Introduction

It is well known that children’s physical fitness (PF) [1] and sedentary behaviors are linked with pediatric obesity levels [2]. It therefore seems reasonable to assume that these behaviors will also negatively affect gross motor coordination (GMC) development, since motor coordination correlates with PF, physical activity (PA), weight status and/or body fatness (BF) during childhood [3,4,5,6]. In addition associations have been found between GMC, body size/shape, PA and PF [7,8]. However, little is known about the extent to which GMC variance may be explained by the additive effects of body fatness, PA and PF levels, and other important biological and environmental factors.

GMC is considered an important element of a child’s motor skills, and their cognitive and psychological behaviors [9]. Balanced and controlled movements have also been linked to social interactions and health-related benefits [10,11], which track throughout adolescence [12]. Children GMC status results from a complex interaction of biological and maturational factors [9,13], influenced by both genes and the environment [14,15]. It has been previously shown that GMC variability is dependent on gender and chronological age (biological factors), especially during the post-pubertal years [13]. Furthermore, fetal life has also been shown to be a contributing factor to motor development across the lifespan, especially during childhood [16]. Low birth weight is considered a strong risk factor for delayed motor development across the lifespan [16]. Environmental factors are also associated with GMC development [14]. For example, the school setting plays a key role in GMC development given the fact that the school is the most highly organized and structured institution within any society [17] where children spend the most part of their daily life. During planed and organized physical education (PE) classes, children’s motor learning and development are influenced in many ways. School routines also increase the options for other organized and informal motor experiences [6,17]. Thus, it is expected that children’s motor development process be adequately addressed during their school attendance, providing them with time for unstructured play and games participation in high quality PE.

Since children GMC development with age is not independent of the school setting, the systematic use of individual and contextual factors could provide the rational as to why children differ in their GMC development. This is an important consideration given that children motor development trajectories will likely be of great relevance to their long-term health-related habits and behaviors. However, the current literature is limited with regards studies that have combined information of age, sex, PF, PA, BF, birth weight and the school environment in a single analysis. Thus, the aims of this paper are to identify child and school level characteristics that explain inter-individual differences in GMC development. The paper addresses the following hypotheses: (1) boys and older children are better coordinated than girls and younger children; (2) the most active children will be better coordinated than the least active; (3) physically fit children will be better coordinated than their less fit peers; (4) low birth weight will impair motor coordination; and (5) school characteristics will significantly affect GMC development.

## 2. Material and Methods

### 2.1. Sample

A cross-sectional sample of children aged 6 to 10 years (186 boys and 204 girls) was recruited from the *Active Vouzela* study. In brief, the *Active Vouzela* study investigated different aspects of children’s and adolescents’ physical growth, maturation, development and parental influences associated with health outcomes [18,19]. Data were collected in 2010. The sample represented about 90% of the public school population of Vouzela, a central region of Portugal, at that time. All study protocols were approved by the Ethics Committee of the Faculty of Sport, University of Porto, school directors, and the Vouzela Health Center. Informed consents were obtained from all parents and/or legal guardians of the children.

### 2.2. Anthropometry

Measurements were made in accordance with the International Society for the Advancement of Kinanthropometry protocols. Stretched stature, with head positioned to the Frankfurt plane, was measured to the nearest 1 mm using a portable stadiometer (Holtain Ltd, UK). Body mass (kg) and total body fat (BF; kg) were assessed using a bio-impedance scale (TANITA BC-418 MA Segmental Body Composition Analyzer, Tanita, Corporation, Tokyo, Japan) with a precision of 0.1 kg. This form of BF measure has been validated previously against Dual-energy X-ray Absorptiometry (DXA) [20].

### 2.3. Gestational Information

Gestational data was obtained from a mother’s interview questionnaire and birth weight from the child’s health booklet.

### 2.4. Physical Activity

Physical activity (PA) was estimated using the Godin and Shephard questionnaire [21], a reliable and valid instrument [22,23] that has been consistently applied to Portuguese children [24,25,26]. Using direct interview the questionnaire assessed aspects of the children’s daily routines; applied by the same team member of the *Active Vouzela* project. Variables recorded included the number of times/week that they spent in different activities for a period of 15 minutes or more. These recorded values were converted to METs. Physical activities were classified into three categories: low (3 METs, e.g., easy walking, touring, playing); moderate (5 METs, e.g., fast walking, leisurely bicycling, non-competitive swimming); and high (9 METs, e.g., running, jogging, soccer, martial arts practice, vigorous swimming). The frequency (number times/week) of each category was multiplied by the respective MET value (METs/week). These three products’ categories were summed to obtain a final score of total PA (TPA), expressed in METs/week.

### 2.5. Physical Fitness

Physical fitness (PF) was assessed by a set of tests using the AAHPER’s *Youth Fitness Test* and *Fitnessgram*. Tests included: agility and velocity (agility shuttle-run and 50-yard dash); lower limbs explosive strength (standing broad jump); hand static strength (handgrip) of dominant hand; and cardiorespiratory fitness (1-mile run/walk test). These tests have been consistently used to assess PF levels in pediatric populations [27,28,29]. All test results were normalized by comparing observed values to sex and age specific group means and standard deviations to generate z-scores. Theses individual test z-scores were summed to compute a global continuous PF score (PFS). Z-score signs were determined by each performance metric to ensure positive results. For example, in a 50-yard dash, those who ran faster had a negative z-score which was later transformed to a positive sign when used for summation. To avoid age z-scores means being equal to zero, the total sample of reference children was used to compute individual z-scores.

### 2.6. Gross Motor Coordination

Gross motor coordination (GMC) was assessed using the *Köperkoordinationtest für Kinder* battery (KTK) of tests. These were developed by Schilling and Kiphard [30] and have been used extensively in Portuguese populations [31]. The assessment comprises four tests: (i) walking backwards on a beam (WB), (ii) hopping height (HH), (iii) jumping sideways (JS), and (iv) moving sideways on boxes (MS). A more detailed description can be found in Vandorpe *et al.* [32]. In the present paper, unweighted sum of scores are reported from the four KTK tests as a measure of total GMC (TGMC), as advocated by Schilling [33].

### 2.7. School Environment

School context information was obtained from a structured inventory. This inventory was developed by the authors in collaboration with the Vouzela city-hall education department. Aspects considered included: school size (number of students); setting (rural, urban and semi-urban area) determined by Portuguese Institute of Statistics; available area for recess time; school infrastructure (sport and PA facilities); frequency of PE classes; and professional qualification of PE teachers. Small differences are related to PA/sport facilities, *i.e.*, schools located in more central region are larger and tend to show more PA and sports facilities than schools located in more rural locations. Since all teachers had the same professional qualifications and all schools provided the same amount of weekly classes, as well as the same curriculum activities and materials (information provided by the City Hall Sports Department), no differences were found in these aspects. Therefore these variables were not considered in the analysis.

### 2.8. Data Quality Control

Data quality control was assessed in three steps: (1) training and careful supervision of all team members; (2) retesting a random sample of 70 children two weeks apart, including the PA questionnaire; and (3) and computing reliability estimates. The technical error of the measurement was 2 mm for stretched stature, and 100g for body mass and BF; intra-tester ANOVA-based intraclass correlation coefficients (R) were 0.80 for TPA, for GMC individual tests the values ranged from 0.84 (MS) to 0.91 (WB), and from 0.81 (agility shuttle-run) to 0.97 (handgrip) for PF tests, which show the high reliability of the collected data (all R ≥ 0.80).

### 2.9. Statistical Analysis

Exploratory data analysis was performed to identify input data errors and outliers, as well as to obtain descriptive information. An independent t-test was used for comparisons between sexes. Modeling the association of children’s TGMC and their individual characteristics (level-1) within the school environmental factors (level-2) was performed using HLM software (version 6) [34].

A sequence of nested models was developed. The deviance between models was used as a measure of global fit; as previously advocated for use in multilevel model research [35,36]. Differences in deviance are distributed as an approximate chi-square (χ^2^) distribution with degrees of freedom equal to the difference in the number of estimated parameters between two models. Predictor variables variance in children TGMC was assessed with a pseudo-R^2^; defined as the proportional reduction in variance for that parameter estimate resulting from the comparison of a new model with the previous one [34]. Modeling was done in a stepwise fashion. First, a null model (M_0_) was estimated to compute the intracluster correlation coefficient, allowing for the estimation of the variance accounted for by the school effect in TGMC. Secondly, two models were developed that included estimates of childhood predictors of TGMC; the first model (M_1_) used only age and sex; the second model (M_2_) estimated the additive effects of age, sex, birth weight, BF, TPA and PFS. These models are called level-1 or student models and tested our Hypotheses 1 to 5. To facilitate the interpretation of these predictors, all but sex (females = 0 and males = 1) and PFS (expressed as a z-value), were centered around their grand means [34]. Lastly, the level-2 model, or contextual model (M_3_), was estimated using school effects and significant predictors of children’s TGMC from model 2 (M_2_), which tested our Hypothesis 5.

## 3. Results

Table 1 shows the descriptive statistics (mean ± SD) for individual-level variables. Height and body mass mean values were similar between sexes, as well as TPA levels (*p* > 0.05). Girls had higher mean BF (*p* < 0.05). Boys performed better in all PF tests (*p* < 0.05), as well as in HH and MS GMC tests (*p* < 0.05). Schools characteristics (Table 2) indicate that all schools had graduated PE teachers, offered the same opportunities for individual and/or team sports, that the frequency and quality of PE classes were the same, and that most schools settings were rural.

**Table 1:** Descriptive statistics for all variables at student level (level I).

Student Level (n = 390)	Girls Mean ± SD	Boys Mean ± SD	*t*	*p*
Age (years)	8.61 ± 1.28	8.37 ± 1.26	−1.83	0.07
**Anthropometry**				
Height (cm)	131.48 ± 8.52	131.24 ± 8.74	2.79	0.78
Body mass (kg)	31.98 ± 7.91	31.26 ± 7.77	0.90	0.37
Birth weight (g)	3270.2 ± 453.63	3386.0 ± 474.14	−2.16	0.03
**Body composition**				
BF (kg)	8.16 ± 3.43	6.80 ± 3.59	3.82	<0.01
**Physical Activity**				
TPA (METs/week)	104.09 ± 47.76	112.55 ± 51.30	−1.68	0.09
**Motor coordination**				
WB (points)	40.54 ± 14.92	37.57 ± 15.78	1.82	0.07
HH (points)	14.92 ± 8.22	18.24 ± 9.79	−3.59	<0.01
JS (points)	42.35 ± 14.14	41.33 ± 12.59	0.75	0.46
MS (points)	34.14 ± 6.96	36.12 ± 7.37	−2.70	<0.01
TGMC (points)	131.30 ± 34.02	133.86 ± 33.76	−0.71	0.48
**Physical Fitness**				
1-mile run (min)	11.47 ± 1.88	10.10 ± 2.32	6.05	<0.01
Standing broad jump (cm)	111.60 ± 20.32	121.00 ± 20.79	4.49	<0.01
Handgrip (kg)	13.32 ± 3.98	14.28 ± 3.84	2.41	0.02
Shuttle-run (s)	13.25 ± 1.50	12.65 ± 1.44	−3.94	<0.01
50 yard-dash (s)	9.83 ± 1.17	9.32 ± 1.18	−4.10	<0.01

BF: total body fat, TPA: total physical activity, WB: walking backwards, MS: moving sideways, HH: hoping for height, JS: jumping sideways, TGMC: total gross motor coordination (sum of all test scores).

Results of the variance component models are presented in Table 3. In the null model (M_0_: deviance = 3436.45), the variance estimate (τ_00_ = 101.60 ± 56.55) at the school level (level 2) was statistically significant (χ^2^(17) = 46.75, *p* < 0.001), indicating a significant inter-individual differences in TGMC development between schools. The intracluster correlation coefficient (ρ) was 0.096 (ρ = 101.60/(101.60 + 1058.86)), *i.e.*, 9.6% of the total variance in TGMC was explained by the school effects, and thus individual children characteristics explained the major fraction of total variance, *i.e.*, 90.4%.

**Table 2:** Descriptive statistics for all variables at school level (level II).

School Level (n =18)	Mean ± SD	Min-Max
**School size (n of students)**	25.67 ± 22.03	7–86
		**n (%)**
**School setting**	Rural	17 (94.4)
	Semi-urban	1 (5.6)
**Playground area**	With playing facilities	16 (88.9)
**Other infrastructure**	Having a sport center	2 (11.1)
	With non-paved outdoor ground	17 (94.4)
	With paved sport ground	3 (16.7)
**Frequency of PE class**	2 times per week	18 (100)
**Time of PE class**	≤45 min	18 (100)
**Qualification of PE responsible**	Graduated in PE	18 (100)

PE: Physical Education.

Finding from the first student model (M_1_), which only considered age and sex as predictors of children’s TGMC, showed that older children were more coordinated (β = 12.43 ± 1.28, *p* < 0.001), but that there was no significant sex effect (β = 3.98 ± 3.14, *p* > 0.05). M_1_ deviance dropped to 3352.95 from M_0,_ indicating an improvement in model fit when age was added (χ^2^ (2) = 83.51, *p* < 0.001, a drop of 83.5 for a loss of 2 degrees of freedom). The second student model (M_2_) included all the other childhood predictors. This second model showed that boys were more coordinated than girls (β = −13.48 ± 3.19, *p* < 0.001), when TGMC were adjusted for age, birth weight, BF, TPA and PFS. Furthermore M_2_ also shows that children who were more physically fit had higher adjusted TGMC values (β = 5.82 ± 0.63, *p* < 0.001) when the other covariates were controlled. The model further showed that individuals who had more fat mass were less coordinated (β = −1.23 ± 0.48, *p* < 0.012). Birth weight (*p* > 0.05), and TPA (*p* > 0.05) were not significant independent predictors of TGMC development; age (*p* = 0.07) showed a marginal trend. M_2_ deviance was 2222.42 (a decrease of 1130.5 for loss of four degrees of freedom), indicating an improvement in model fit between models M_2_ and M_1_ (χ^2^ (4) = 1130.52, *p* < 0.001). The proportion of level-1 variance (within-school) explained by students’ characteristics in TGMC was 62.6%

The contextual model assessed the magnitude and significance of school effects. In this model, all non-statistically significant level-1 variables (TPA and birth weight) were excluded. An increase in M_3_ deviance was found when school fixed effects were included in the model. School size (student numbers) has a significant negative independent effect on children’s TGMC (β = −0.39 ± 0.1 ± 1, *p* = 0.005). Paved sport ground was a facilitator of higher motor coordination levels (β = 14.266.73, *p* = 0.05). The remaining infrastructure variables were not statistically significant predictors of TGMC. The reduction in the variance component at the school level was from 101.60 in the null model (M_0_) to 17.41 in contextual model (M_3_). The proportion of between-school variance attributed to significant school variables was 84.2%.

**Table 3:** Summary of results of the three nested models.

Sum of Gross Motor Coordination Score				
Parameters	Null Model (M_0_)	Model I (M_1_)	Model II (M_2_)	Model III (M_3_)
***Regression coefficients (fixed effects)***				
Intercept	131.67 (3.11) ***	130.86 (3.08) ***	140.78 (2.90) ***	144.81 (8.73) ***
Age		12.43 (1.28) ***	3.42 (1.853) *	2.51 (1.59)
Sex		3.98 (3.14)	−13.48 (3.19) ***	−15.880 (2.80) ***
Birth weight			−2.10 (3.25)	
BF			−1.23 (0.48) ***	−1.47 (0.42) ***
TPA			−0.03 (0.03)	
PFS			5.82 (0.63) ***	6.45 (0.56) ***
School size				−0.39 (0.11) ***
Type of playground area				4.18 (6.05)
Having a sport center				4.34 (5.83)
With non-paved outdoor ground				−5.69 (6.88)
With paved sport ground				14.27 (6.73) **
***Variance Components (random effects)***				
School mean	101.60 (56.55)	67.41 (40.01)	52.51 (31.56)	17.06 (16.14)
Children level effect	1058.86 (82.17)	837.81 (65.00)	482.73 (45.20)	506.08 (41.76)
***Model summary***				
Deviance	3436.45 ***	3352.95 ***	2222.42 ***	2806.75 ***
Number of parameters	3	5	9	12

Standard errors are in parenthesis; *** *p* < 0.01; ** *p* < 0.05; * *p* = 0.07; BF: total body fat, TPA: total physical activity, PFS: physical fitness score.

## 4. Discussion

Our findings suggest that school effects explain approximately 10% of the variance in children’s TGMC development, with the child’s individual characteristics accounting for the other 90% of variance. Sex, PFS and BF were all found to be important child-level predictors of TGMC development; school size had a negative effect on children’s TGMC development, while paved sport grounds facilitated high TGMC levels.

Similar sex differences in GMC have been previously reported [25,37,38], indicating that boys, at comparable ages, are more coordinated than girls. This suggests that such findings are due to differences in motor skills refinements, body growth and PF levels. We only found sex differences in Models 2 and 3; in the last model (M_3_) no significant effects of age were identified. It is important to understand that age was only significant when PFS and BF were not included in the models. Possibly, the age effect is jointly confounded by total PF and BF, *i.e.*, as these variables conjointly change as children grow [13] and may blunt the age effect on TGMC development. Although previous studies showed changes in GMC with age, their analysis failed to consider the joint effects of other possible covariates, namely PF and body composition, which were included in the present study [30,31,32] and may explain the observed differences.

Positive associations between PFS and TGMC, as well as, an inverse relationship between BF and TGMC were also observed, suggesting that children who were more physically fit were also better coordinated. Although few studies have explored such associations independently [4,7,37], most highlight the positive association of PF and GMC; which is linked with motor skills refinements. It is also suggested that children with poor motor proficiency also have lower PF and have higher percent BF when compared to their peers with adequate GMC levels [39]. Children with motor difficulties usually find simple motor tasks challenging, thus decreasing PF development and associated motor coordination [39,40]. This leads to an energy imbalance and influences BF accumulation; especially if unhealthy eating behaviors are also present. Although this complex relationship is more clearly seen in poorly coordinated children, it has also been observed in those with adequate motor coordination as well [6].

Previous studies have shown an inverse relationship between birth weight and motor proficiency, although the motor deficits’ consequences depend on the severity of fetal development adverse conditions. Datar and Jacknowitz [41] observed a catch-up effect in motor development in low birth weight children, not observed in extremely low birth weight children [42]. In the present study we did not find any birth weight effects on TGMC development, likely due to the fact that low birth weight (<2500 g) frequency in our sample was less than 3.6% (n = 11). It is also possible that different motor opportunities during early childhood may have reduced and/or annulated the negative consequences of low birth weight.

We did not find any physical activity effects associated with GMC development. According to Holfelder and Schott [5], only a few studies have examined possible “causal” relationships between motor coordination and PA [43,44]; finding have shown that this link has not yet been sufficiently clarified in terms of effect size or directional path. For example, previous studies suggested that GMC was an important predictor of PA [31,45]. Notwithstanding, the direction of this relationship may be mediated by age [5,40], *i.e.*, in early childhood physical activity opportunities are essential to develop good GMC levels; during middle and late childhood, as well as, in adolescence, GMC level differences become more evident between children and may in turn affect their physical activity participation [45]. This supposition posits that GMC levels are the basis, rather than the consequence, of PA changes, indicating that longitudinal studies in natural and/or experimental contexts are required to clearly elucidate this complex issue [46].

Of the school variables included in the models only school size and paved sport ground had independent significant effects on GMC development. School size expresses global school dimensions and facilities. In the present study, school size was negatively related to GMC. As the majority of children were from smaller rural schools and it is possible that this environment provided more opportunities for motor skills development; at the same time their school based physical games and play repertoire may have induced greater GMC [47]. In contrast, paved sport grounds were positively related to GMC. These grounds were used for both PE classes and free playtimes. Empirical findings suggest that children’s behavior on the play area is influenced by the delineation of space, degree of challenges, novelty, and complexity [48], and different opportunities to motor skill development which determine children’s motor development [32,49].

In the present study, school characteristics did not show variability between schools. The Vouzela region is predominantly rural, where PA/sport facilities and road access diversity are similar. Small differences are related to the availability of sport centers and paved sports grounds. The majority of these schools are located in more rural regions, showing smaller spaces than more central schools. Notwithstanding, City-Hall policy provides equal conditions for motor learning and diversified PA practices within the school system. In addition, school-based curriculum in Portuguese primary schools are applied in the same way in all schools, comprising two times per week of PE classes, in addition to other activities programs all supervised by PE teachers. In the Vouzela region, the frequency, curriculum organization and quality of PE classes is the same for all schools.

Potential limitations of the study include sample size at the student and school levels because they influence the accuracy of all parameter estimates as well as their standard errors [35]. However, Maas and Hox [50] argued that regression coefficient estimates are unbiased even if the sample size is small. Although subjective measures of PA have been questioned, the Godin and Shephard questionnaire used has been shown to be highly reliable in different sub-samples of Portuguese children [24,25]. In our models, children were nested within schools. As such, classes were missed in this link, although we were not able to localize any study that used “class climate” markers as suitable predictors of GMC. Furthermore, none of the available studies aiming at interpreting children GMC development considered nesting effects. In any case, since we adjusted children GMC levels by their school nesting, we do not anticipate any errors in the statistical tests of our parameter estimates (*i.e.*, regression coefficients). Lastly, information concerning children’s home environments was not included in the present analysis. We were not able to locate published data-driven studies where the importance of home environmental factors enhancing GMC were elucidated with children from 6–10 years of age, although home factors may be associated with children’s motor development during early childhood [49,51,52].

The advantages of the approach used include: (i) using a single coherent multilevel framework to address the complex issue of individual-level and school-level variables. (ii) Secondly, a wide array of valid and highly reliable data concerning growth, body composition, and motor performance was used in disentangling TGMC variance. Taken together, this is the first time that such an attempt was made using multilevel modeling with an extensive list of TGMC variables in children.

## 5. Conclusions

The present results showed that individual children characteristics explained 90% of TGMC variance, of which 62.6% was accounted for the independent additive effects of age, sex, BF and PF. As expected with increasing age children were better coordinated, boys outperformed girls, and those with higher fat mass levels were less coordinated. School variables explained 84.2% of the 10% variation fraction attributable to school level. Although the school environment seemed to play a minor role in GMC development, it is very important to stress the relevance of the school context and climate in providing adequate opportunities for children’s motor development; namely high quality PE classes and sufficient infrastructure conditions for organized and non-organized physical activities. This suggests that the school may be the best place to apply effective sport-based school programs to prevent overweightness and obesity, the best place to improve PF levels, and influence GMC development.
